# All-solid anti-resonant single crystal fibers

**DOI:** 10.1007/s12200-022-00003-w

**Published:** 2022-03-29

**Authors:** Jinmin Ding, Fanchao Meng, Xiaoting Zhao, Xin Wang, Shuqin Lou, Xinzhi Sheng, Luyun Yang, Guangming Tao, Sheng Liang

**Affiliations:** 1 Key Laboratory of Education Ministry on Luminescence and Optical Information Technology, National Physical Experiment Teaching Demonstration Center, Department of Physics, School of Science, Beijing Jiaotong University, Beijing, 100044 China; 2 School of Electronic and Information Engineering, Beijing Jiaotong University, Beijing, 100044 China; 3 Wuhan National Laboratory for Optoelectronics, Huazhong University of Science and Technology, Wuhan, 430074 China; 4 State Key Laboratory of Crystal Materials, Shandong University, Jinan, 250100 China

**Keywords:** Single crystal fiber (SCF), Anti-resonant (AR) optical fiber, Few-mode fiber, Modal reduction, Confinement loss, Finite element method

## Abstract

In this paper, a novel all-solid anti-resonant single crystal fiber (AR-SCF) with high refractive index tubes cladding is proposed. By producing the cladding tubes with high refractive index material, the AR guiding mechanism can be realized for the SCF, which can reduce the mode number to achieve single-mode or few-mode transmission. The influences of different materials and structures on the confinement loss and effective guided mode number for wavelengths of 2–3 μm are investigated. Then, the optimal AR-SCF structures for different wavelengths are determined. Furthermore, the influences of different fabrication errors are analyzed. This work would provide insight to new opportunities in the novel design of SCFs by AR, which would greatly impact the fields of laser application, supercontinum generation, and SCF sensors.

**Graphical Abstract:**

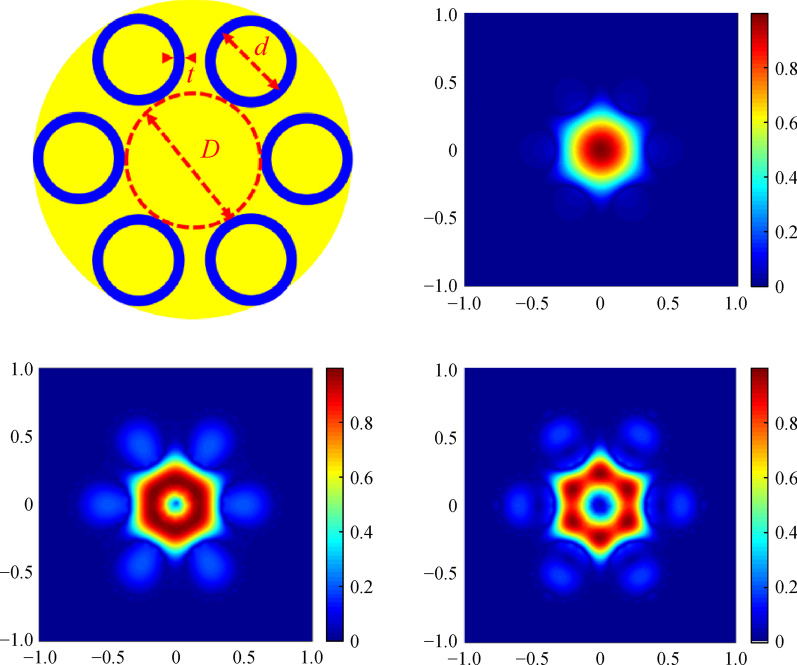

## Introduction

As a combination of bulk crystals and traditional fibers, single crystal fibers (SCFs) inherited the advantages of crystals, including high melting points, high thermal conductivity, high laser damage threshold, and good mechanical properties. They also carried the advantages of conventional optical fibers, such as having a large aspect ratio and specific surface area, both of which remarkably improve their thermal management capabilities [1, 2]. SCFs have been widely used in many fields due to their excellent comprehensive properties. As an outstanding fiber laser gain medium, SCFs are expected to have higher melting points, thermal conductivity, strength, laser damage threshold (500 times higher than that of silica), doping concentration, and a lower Brillouin gain coefficient [3–5]. Compared to silica fiber, sapphire SCF is a better platform for supercontinum generation due to its high transparency up to 5 μm, low material dispersion in the 0.8 –5 μm spectral range, and higher laser damage threshold [6, 7]. Furthermore, SCF-based sensors are the unique and effective solution in high-temperature, high-pressure, and chemically aggressive environments that require high temperature stability and transient response characteristics because of their high melting point, superb mechanical properties, and stable physical and chemical properties [8–11].

However, the SCFs are multi-mode without cladding, which leads to significant difficulties during application. Therefore, SCF cladding has been utilized to reduce the confinement loss (*CL*) and the number of effective guided modes to achieve the single-mode or few-mode transmission.

Current SCF cladding techniques are based on the principle of total reflection, also known as refractive index guiding, which is achieved by adjusting the refractive index of the cladding to be lower than that of the core. Therefore, it is common for cladding materials to have lower refractive indices and similar thermal expansion coefficients to that of the core. Effective cladding methods include the magnetron sputtering [12], Sol–Gel method [13, 14], liquid phase epitaxy [15], co-drawing laser heating pedestal growth (LHPG) [16–24], ion implantation [25–28], and micro-structure cladding [7, 29, 30].

The disadvantages of the existing refractive index guiding cladding techniques include the small bandwidth and requirement of high structure accuracy during preparation. However, other light-guiding mechanisms may aid in the potential advancement of SCF cladding. The anti-resonant (AR), or negative curvature, optical fibers with a cladding that consists of a single ring of tubes have sparked a lot of interest due to their low losses over a wide wavelength span, simple cladding structure, and freedom in design [31, 32].

In this paper, by designing an analogy of current air-glass AR hollow-core fiber structure, we have proposed and investigated an all-solid AR-SCF. The AR guiding can shed light on new opportunities for cladded all-solid SCFs with simple cladding structure, a potential SCF design with low *CL* and single or few mode transmission.

## Design of anti-resonant single crystal fiber (AR-SCF)

### Structures

**Fig. 1:**
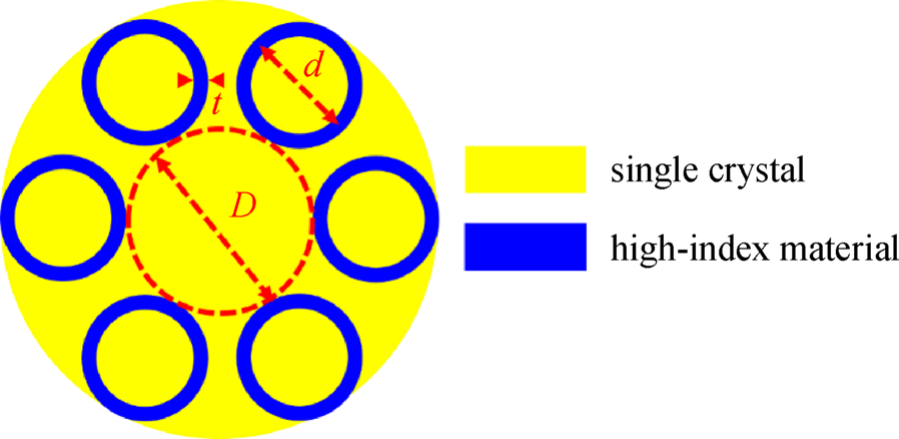
Structure of all-solid AR-SCF with single-ring cladding tubes. *D*: diameter of core, *d*: diameter of the cladding-tube, and *t*: thickness of the cladding-tube

Our proposed all-solid AR-SCF with single-ring tubes is shown in Fig. 1. As an analogy of the air-glass AR optical fiber structure, a single ring of the high refractive index material tubes (blue in Fig. 1) with diameter *d* and thickness *t* is embedded in the single crystal material (yellow in Fig. 1). The area in the center of the tubes is the core with the diameter *D*.

### Materials

The optical and thermal parameters of the single crystal core and cladding materials are shown in Table 1.

**Table 1:** Materials of the single crystal core and cladding tubes

Materials		Refractive index@2.8 μm	Melting point/°C
Single crystal	Sapphire (Al_2_O_3_)	1.7490–1.7170	2054
	Lutetium oxide	1.901	2510
	Y_2_O_3_	1.90–1.87	2439
	MgAl_2_O_4_	1.703–1.668	2250
	SiC	2.58–2.52	2830
	Nd:YAG	1.81	1970
	LiNbO_3_	2.236–2.1615	1253
	BaB_2_O_4_	1.6556–1.6129	1060
Cladding-tubes	Lanthanide glass (S-LAN79)	2.0008	699
	AsS	2.35	180
	GeAsS	2.22	420
	GeS	2.11	380
	ZnS	2.19	1830
	As_2_S_3_	2.4218	197

## Results and discussion

### Numerical method

The AR-SCF was numerically investigated using the finite element method (FEM) with the circular perfectly matched layer (PML), which allowed us to calculate the value of *CL* according to the equations [33]:

1 $$CL = 8.686\times k\times{\text{Im}}\left( n _{\text{eff}} \right),$$

2 $$k=\frac{2\uppi}{\lambda},$$

where Im(*n*_eff_) is the imaginary parts of *n*_eff_, *λ* is the wavelength.

For the step index fiber, the number of guided modes can be calculated using the following equations:

3 $$V=\uppi \frac{d}{\lambda }NA,$$

4 $$NA=\sqrt{{{n}_{1}}^{2}-{{n}_{2}}^{2},}$$

5 $$M=\frac{4}{{\uppi }^{2}}{V}^{2},$$

where *d* is the fiber diameter, *n*_1_ and *n*_2_ are the refractive indices of the core and cladding tube, *NA* is the numerical aperture, and *M* is the number of modes. However, this method is ineffective for the AR-SCF due to significant errors calculated by Eq. (5). Therefore, we determined the number of guided modes by calculating the effective refractive and the *CL* difference between the higher order mode and the fundamental mode. If the *CL* of the higher order mode is three orders of magnitude greater than the *CL* of the fundamental mode, then the higher order mode is considered a less effective transmission mode.

### Numerical results

The light-guiding in AR fibers is achieved by inhibited coupling between the core and cladding modes. The low loss transmission windows are determined by the cladding capillary thickness, according to the anti-resonant reflecting optical waveguide (ARROW) model. Then, one of the most important performances of AR-SCF is the *CL*, which relates to the leaky nature of the guided modes, is the dominant loss mechanism in the near infrared region.

**Fig. 2:**
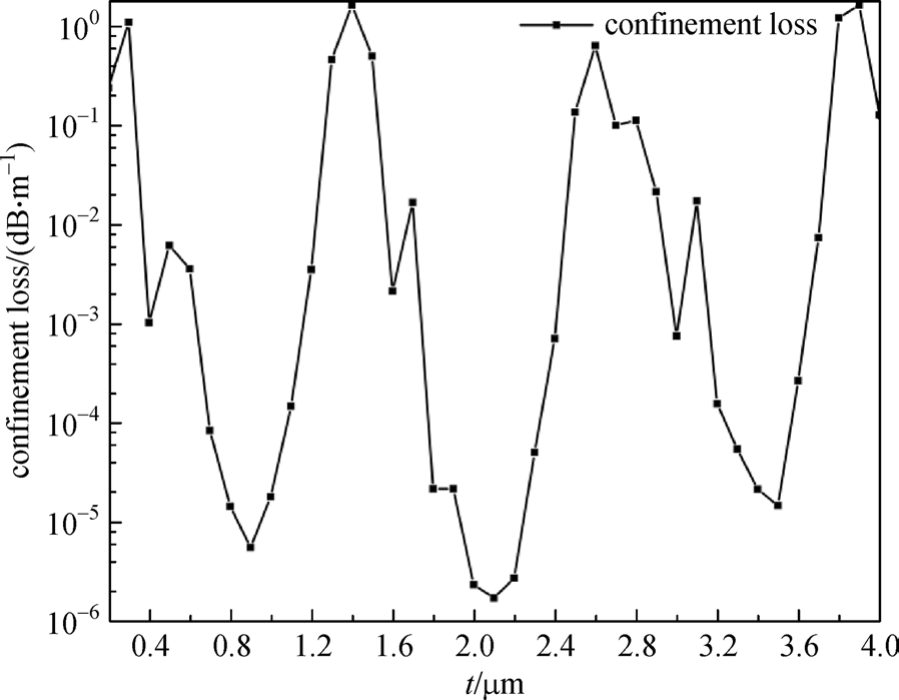
Confinement loss (*CL*) with the thickness (*t*) of cladding-tube at wavelength of 2.8 μm. *n*_1_ = 1.89, *n*_2_ = 2.20, *D* = 30 μm, *d* = 20 μm

First, in order to verify the anti-resonant light-guiding of AR-SCF, the *CL* with the thickness of cladding-tubes (*t*) from 0.2 to 4.0 μm at wavelength of 2.8 μm is shown in Fig. 2, with the structure parameters *n*_1_ = 1.89, *n*_2_ = 2.20, *D* = 30 μm, *d* = 20 μm.

**Fig. 3:**
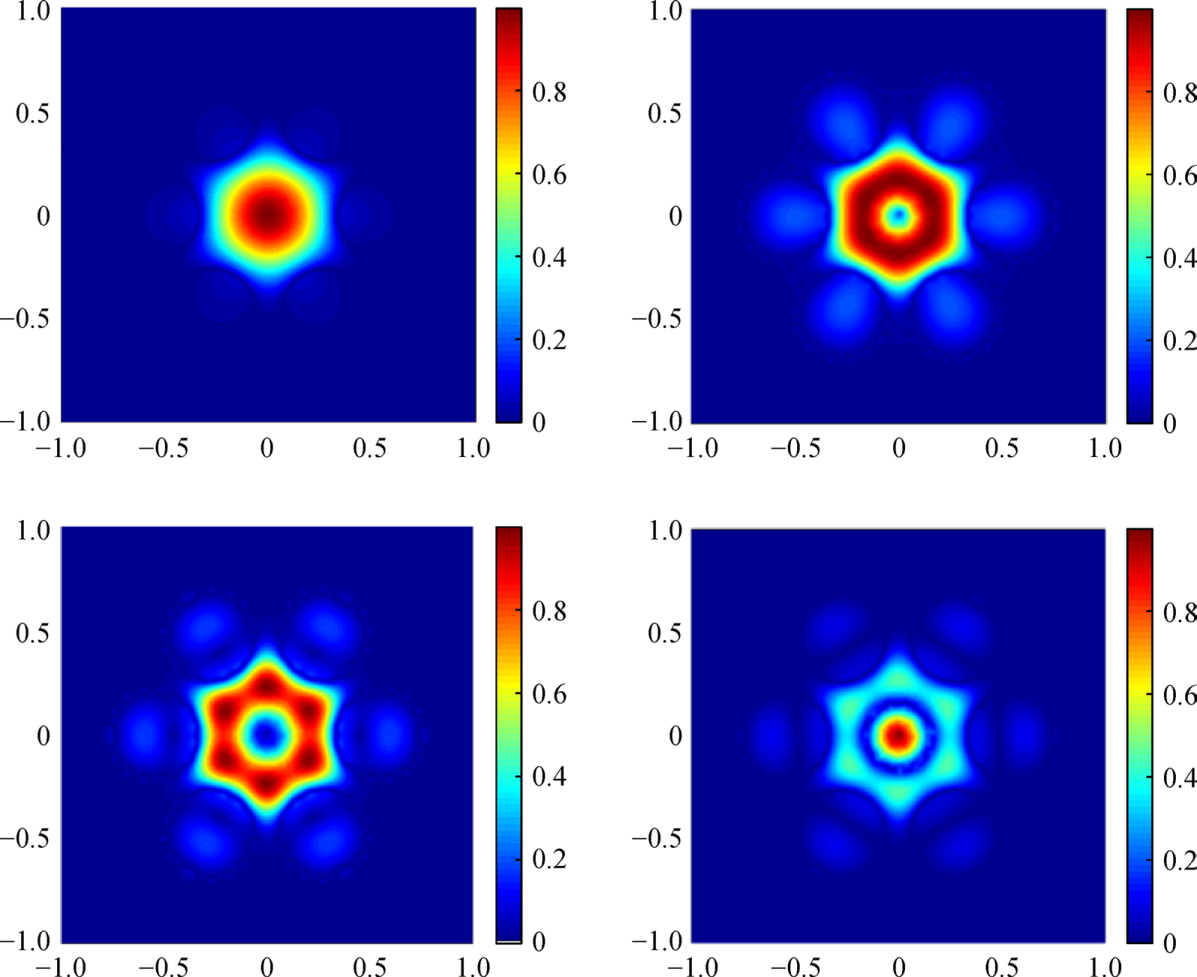
Normalized intensity profiles of the AR-SCF. *n*_1_ = 1.89, *n*_2_ = 2.20, *D* = 30 μm, *d* = 20 μm, *N* = 6, and *t* = 0.9 μm, at wavelength of 2.8 μm

As exhibited in Fig. 2, with the increase of *t*, the low *CL* changes periodically, namely characteristic of anti-resonant light-guiding. It is the anti-resonant state when *t* is 0.9, 2.1, and 3.5 μm, with *CL* less than 1 × 10^−4^ dB/m. When *t* is 0.3, 1.4, 2.6, and 3.9 μm, and *CL* is higher than 1 dB/m, the corresponding thickness of the cladding-tubes are ineffective in the achievement of light-guiding. There are four guide modes at the anti-resonant point and the normalized intensity profiles of the AR-SCF when *t* is 0.9 μm and the wavelength is 2.8 μm are shown in Fig. 3. The above results suggest that the all-solid AR-SCF can be achieved.

**Fig. 4:**
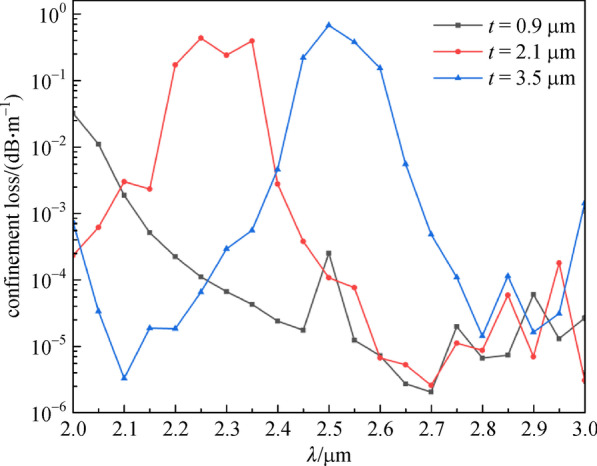
Confinement loss (*CL*) with wavelength for different cladding-tube thickness (*t*). *n*_1_ = 1.89, *n*_2_ = 2.20, *D* = 30 μm, *d* = 20 μm

Figure 4 shows the relationship between *CL* and wavelength (range is 2–3 μm) for different *t*. When *t* is 0.9 μm, the value of *CL* initially decreases as the wavelength increases, then gradually increases after 2.7 μm. When *t* is 2.1 μm, the *CL* initially increases, then decreases, and finally increases again when the wavelength increases from 2.0 to 3.0 μm. The trend of *CL* adds an additional decrease when the wavelength increases from 2.0 to 2.1 μm, compared to when *t* is 2.1 and 3.5 μm. No obvious changes in *CL* are observed when the wavelength is 2.8 μm with different *t*, though the lowest *CL* can be obtained when *t* = 0.9 μm.

**Fig. 5:**
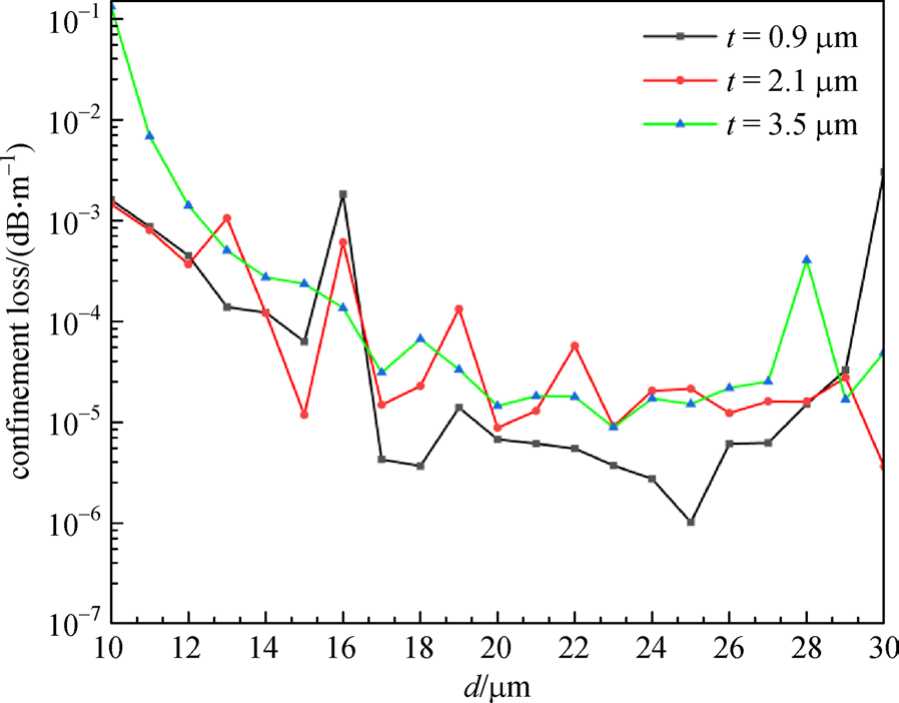
Confinement loss (*CL*) with cladding-tube diameter for different cladding-tube thickness (*t*) at wavelength of 2.8 μm. *n*_1_ = 1.89, *n*_2_ = 2.20, *D* = 30 μm, *d* = 20 μm

The relationship between *CL* and *d* in the cases of different *t* is reported in Fig. 5. The *CL* in Fig. 5 decreases as *d* increase, and finally, the decrease appears fairly smooth. The *CL* of *t* = 3.5 μm is larger than that of *t* = 0.9 μm and *t* = 2.1 μm. In the case of *t* = 0.9 μm, the *CL* changes most gently with the *d*. The lowest *CL* appears at *t* = 0.9 μm and *d* = 25 μm. Therefore, the optimal structure of all-solid AR-SCF can be determined to be as follows: *n*_1_ = 1.89, *n*_2_ = 2.20, *D* = 30 μm, *d* = 25 μm, *N* = 6, and *t* = 0.9 μm, with the *CL* of 1.00623 × 10^−6^ dB/m, and number of guided mode of 4.

It is found that the value of *d* has no remarkable effect on the *CL* compared to the value of *t*. With that in mind, the design of the *d* can focus on the feasibility of fabrication.

**Fig. 6:**
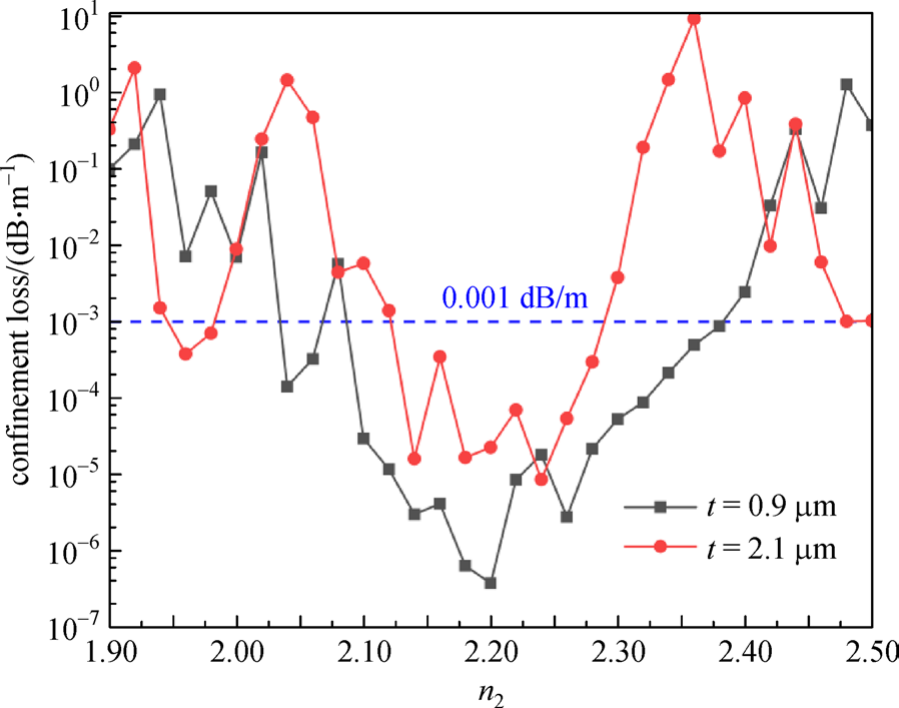
Confinement loss (*CL*) with cladding-tube refractive index (*n*_2_) for different cladding-tube thickness (*t*) at wavelength of 2.8 μm. *n*_1_ = 1.89, *D* = 30 μm, *d* = 25 μm

To investigate the influence of different cladding tube materials, Fig. 6 presents the relationship between the *CL* and the cladding-tubes refractive index (*n*_2_), with different cladding-tube thicknesses. With *t* as 0.9 and 2.1 μm, the *n*_2_ varies from 1.90 to 2.50. We also see that the *CL* first decreases, then increases, with the increase of the *n*_2_. The *CL* of *t* = 0.9 μm is lower than that of *t* = 2.1 μm. Considering 0.001 dB/m as the threshold for acceptable *CL* of AR-SCF, we can obtain the range of the *n*_2_ under different *t.* When *t* = 0.9 and 2.1 μm, the ranges of *n*_2_ are 2.06 –2.38 and 2.16– 2.28, respectively. Furthermore, there is little change in the number of guide modes as the value of *n*_2_ varies. With the guide-mode number mainly stable at 4, we can see that *n*_2_ takes on a smaller acceptable range as *t* increases.

**Fig. 7:**
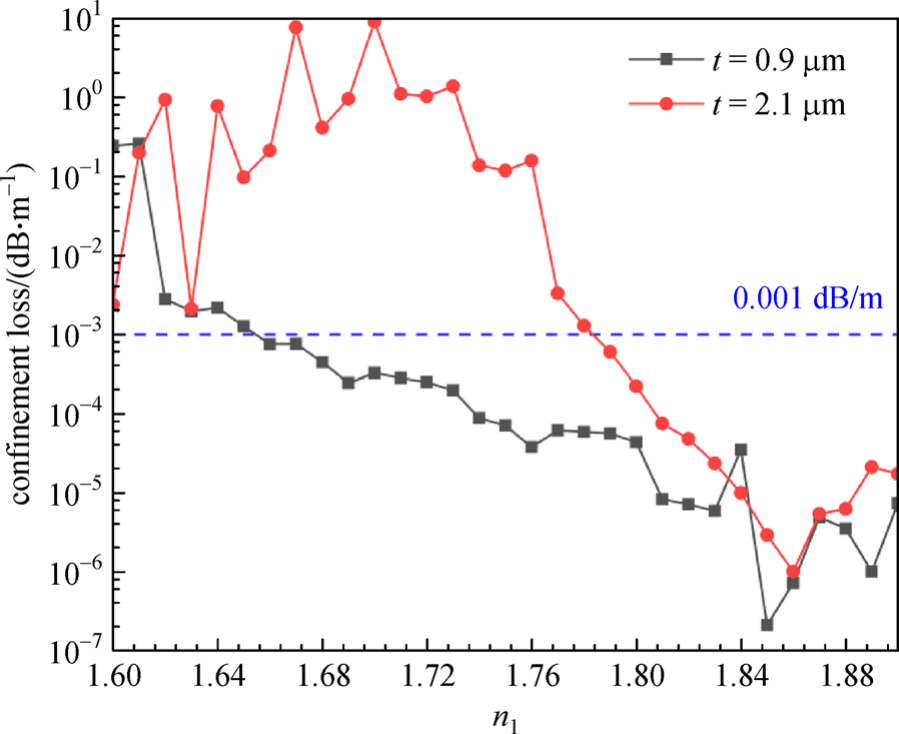
Confinement loss (*CL*) with the core refractive index (*n*_1_) for different cladding-tube thickness (*t*) at wavelength of 2.8 μm. *n*_2_ = 2.2, *D* = 30 μm, *d* = 25 μm

Figure 7 depicts the relationship between the *CL* and the core refractive index (*n*_1_) for different cladding-tube thicknesses. The *t* is 0.9 and 2.1 μm, the *n*_2_ is 2.2, and the variation range of *n*_1_ is from 1.60 to 1.90. We can see that when *n*_1_ ≤ 1.85, the *CL* decreases as the refractive index of the core increases, then increases gradually when *n*_1_ ≥ 1.85. Meanwhile, the overall *CL* of *t* = 0.9 μm is lower than the *CL* of *t* = 2.1 μm, and the number of guide modes is maintained at 4 constantly. If we consider 0.001 dB/m as the threshold, we can determine the value range of *n*_1_ under different *t.* When *t* = 0.9 μm, the *n*_1_ is varies from 1.66 to 1.90; when *t* = 2.1 μm, the *n*_1_ is range from 1.78 to 1.90.

**Fig. 8:**
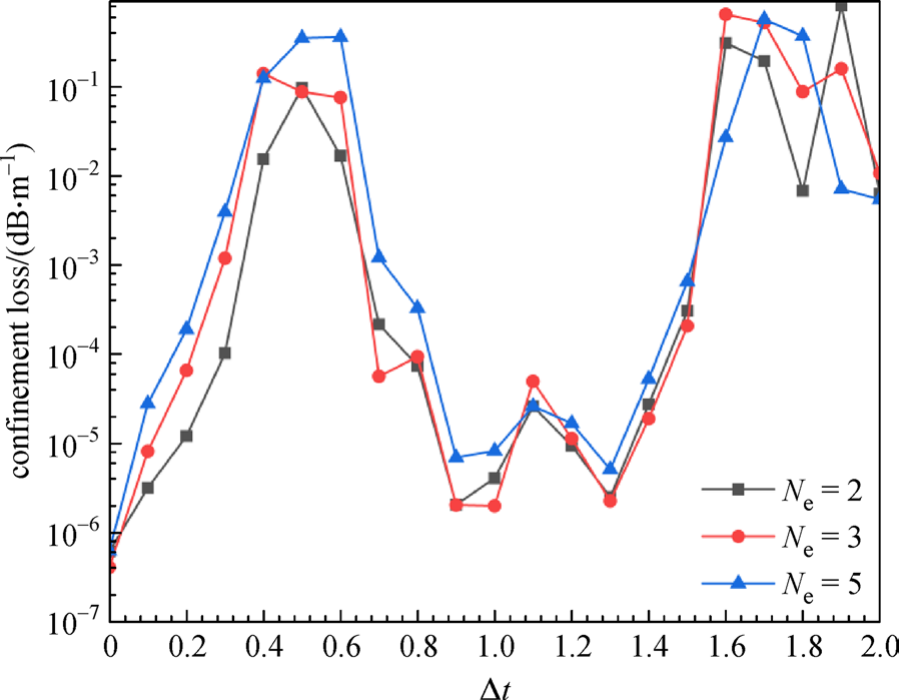
Confinement loss (*CL*) with errors (∆*t*) of cladding-tube thickness for different cladding-tube number (*N*_e_) at wavelength of 2.8 μm. *n*_1_ = 1.89, *n*_2_ = 2.20, *D* = 30 μm, *d* = 25 μm, *t* = 0.9 μm

**Fig. 9:**
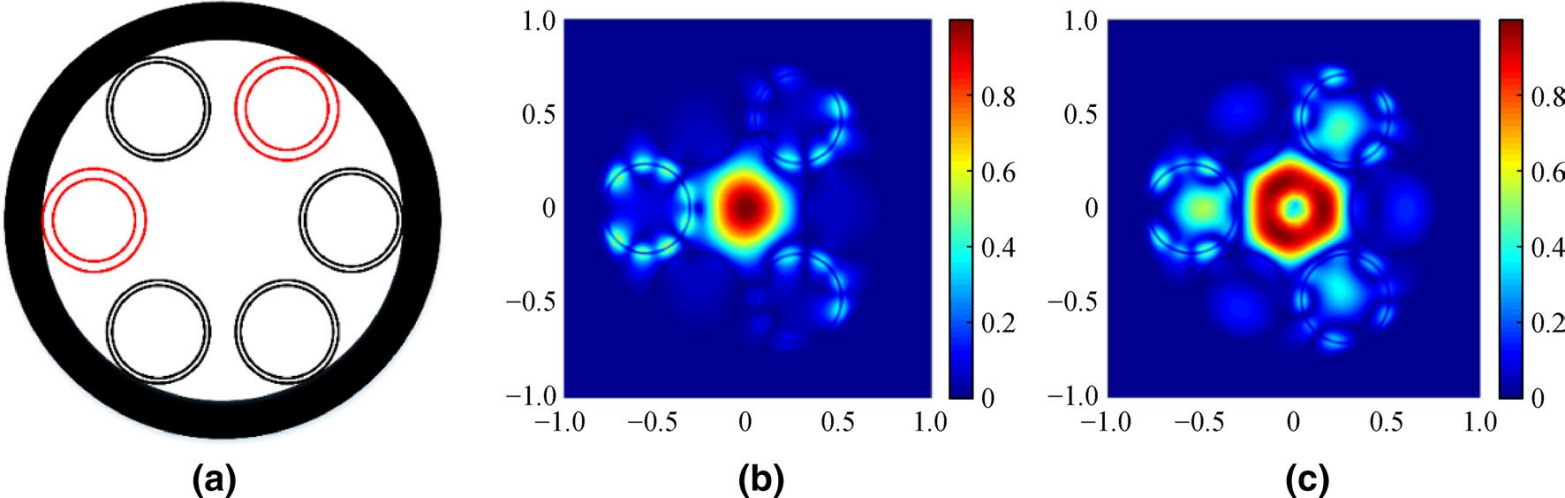
AR-SCF structure with ∆*t* = 0.5 μm, *N*_e_ = 2 **a**, and the normalized intensity profiles of fundamental mode **b** and second order mode **c**. *n*_1_ = 1.89, *n*_2_ = 2.20, *D* = 30 μm, *d* = 25 μm, and *t* = 0.9 μm, at wavelength of 2.8 μm

Next, we analyzed the influence of different fabrication errors on the value of *CL* and number of guided modes of AR-SCF. Figure 8 denotes the relationship between the *CL* and the cladding-tube thickness error (∆*t*). The cladding-tubes that have thickness error are represented in red in Fig. 9a, with *N*_e_ referring to the number of these cladding-tubes.

As ∆*t* varies from 0 to 2.0 μm, the value of *CL* also changes periodically with little difference in *N*_e_, as seen from the curve. However, the ∆*t* greatly influences the AR-SCF mode. Figures 9b and 9c show the normalized intensity profiles of the fundamental and the second order mode when ∆*t* = 0.5 μm and *N*_e_ = 2. From Fig. 9, we can see that the mode field of the fiber changes with the ∆*t*, and the higher order modes changing more drastically. Therefore, the guide mode of the AR-SCF with cladding-tube thickness error is 2.

**Fig. 10:**
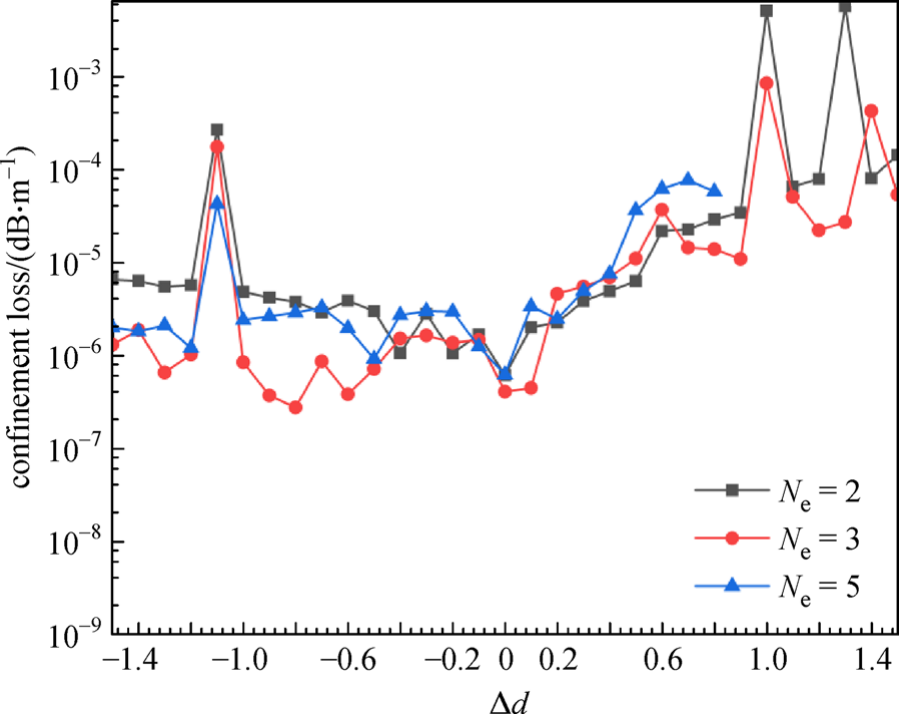
Confinement loss (*CL*) with errors (Δ*d*) of cladding-tube diameter for different cladding-tube number (*N*_e_) at wavelength of 2.8 μm. *n*_1_ = 1.89, *n*_2_ = 2.20, *D* = 30 μm, *d* = 25 μm, *t* = 0.9 μm

Using a similar approach, we analyzed the influence of different cladding-tube diameter errors (Δ*d*) on the *CL* and number of guide modes. Figure 10 illustrates the relationship between the *CL* and the Δ*d* with *N*_e_ = 2, 3 and 5. When the *N*_e_ is 2 and 3, Δ*d* varied from  −1.5 to 1.5 μm. When the *N*_e_ is 5, Δ*d* varied within the range of  −1.5–0.8 μm. The minus sign indicates a smaller diameter and the plus sign indicates a larger diameter. As seen from Fig. 10, the variation trends of the three curves are essentially the same. The *CL* gradually increases as Δ*d* increases, and the *CL* caused by the increase of diameter is greater than the decrease of diameter. Moreover, when the Δ*d* exceeds ±1.1 μm, the *CL* curve will show significant fluctuations. Therefore, we determined that the variation range of Δ*d* is between  −1.1 and 1.1 μm.

**Fig. 11:**
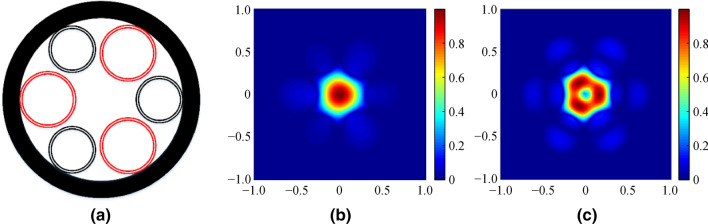
AR-SCF structure with errors of cladding-tube diameter **a**, and the normalized intensity profiles of fundamental mode **b** and second order mode **c**. *n*_1_ = 1.89, *n*_2_ = 2.20, *D* = 30 μm, *d* = 25 μm, and *t* = 0.9 μm, at wavelength of 2.8 μm (∆*d* = 0.5 μm, *N*_e_ = 3)

The effect of the Δ*d* on the guide modes reported in Fig. 11 illustrates the normalized intensity profiles of the fundamental and second order mode, with ∆*d* = 0.5 μm and *N*_e_ = 3. An increase in Δ*d* changes the mode field, especially on the higher order mode, which decreases the number of guide modes from 4 to 2.

### Discussion

The optimal structure parameters that attained by numerical simulation of the AR-SCF is *n*_1_ = 1.89, *n*_2_ = 2.20, *D* = 30 μm, *d* = 25 μm, *N* = 6, *t* = 0.9 μm, the *CL* and guide mode number is 1.00623 × 10^−6^ dB/m and 4, respectively. The fabrication of cladded single crystal fiber has been the main challenge faced during the application of single crystal fibers. However, we can expect to achieve the production of all-solid AR-SCF by improving the existing fabrication technology. The all-solid AR-SCF can be fabricated using the laser processing method. First, the single crystal core can be obtained using the edge-defined film-fed growth method (EFG) [34] and laser heated pedestal method (LHPG) [21]. Then, the cladding ring structure is etched using femtosecond laser processing technology [35]. Finally, the low melting point and high refractive index glass material is injected into the ring structure to prepare the fiber.

## Conclusions

In this paper, we proposed a novel AR guiding SCF with high refractive index tubes cladding. By producing the cladding tubes using high refractive index material, AR guiding can be realized for SCFs, which can reduce the mode number to achieve single-mode and few-mode transmissions. First, we analyzed the feasibility of the anti-resonant light guiding mechanism, and results show that the all-solid single crystal fiber can realize that light guiding mechanism. We then discussed the influence of the AR-SCF structure parameter on confinement loss and guide modes, including the effect of wavelength, cladding-tube diameter, cladding material, and core material. Through our calculations, we determined the optimal structure to be: *n*_1_ = 1.89, *n*_2_ = 2.20, *D* = 30 μm, *d* = 25.μm, *N* = 6, and *t* = 0.9 μm with *CL* = 1.00623 × 10^−6^ dB/m and guide mode number is 4. Finally, we analyzed the influence of fabrication errors on confinement loss and number of guide modes, including the cladding-tube thickness error and cladding-tube diameter error. Additionally, we determined the tolerance range of fabrication error, providing a reference for the actual fabrication. In summary, our work would provide insight to new opportunities in the novel design of SCFs using different light-guiding techniques, which would greatly impact the field of laser application, supercontinum generation, and SCF sensors.
